# Supplementary material for: MIND-NL diet adherence moderates the relation of low-grade systemic inflammation with neuroinflammatory metabolites and cognitive functioning: an exploratory cross-sectional study in older adults
Source: J Neuroinflammation. 2026 Apr 9;23:178. doi: 10.1186/s12974-026-03773-8 (PMC13220514; doi:10.1186/s12974-026-03773-8)
Supplement: Supplementary file 1 — Supplementary Material 1 [file 12974_2026_3773_MOESM1_ESM.pdf]

Supplementary tables

Supplementary Table 1. Modifiable cardiovascular risk factor scale used for study inclusion.

**Risk Factor (self-reported)**	**Point(s)**
BMI ≥ 25kg/m^2 (overweight)	1
Physical inactivity (below the 2020 WHO guidelines: <300 minutes of moderate intensity aerobic physical activity or <150 minutes of vigorous intensity aerobic physical activity per week, spread out over several days)	1
Hypertension (systolic blood pressure ≥140 mmHg, and diastolic blood pressure ≥90 mmHg)	1<br>(2 points are assigned if hypertension is not being actively treated with antihypertensive medication, given the increased cardiovascular burden)
Hypercholesterolemia (total cholesterol >5 mmol/L, or LDL-cholesterol >3 mmol/L)	1
Diabetes type-II	1
Mild cardiovascular disease (e.g. intermittent claudication, varicose veins; In contrast, moderate or severe cardiovascular disease such as stroke, angina pectoris, heart failure, myocardial infarction or revascularization surgery in the last 12 months before pre-screening are exclusion criteria)	1

Supplementary Table 2. Components of the Dutch version of the Mediterranean-DASH Diet Intervention for Neurodegenerative Delay (MIND-NL) diet and cut-off values per score type.

MIND-NL food groups	Score		
	0	0.5	1
Green leafy vegetables	≤29 g/day	>29 - <100 g/day	≥100 g/day
Other vegetables	<71 g/day	≥ 71 -<100 g/day	≥100 g/day
Berries and strawberries	<7 g/day	≥7 - <36 g/day	≥36 g/day
Legumes	<9 g/day	≥9 - <26 g/day	≥26 g/day
Nuts	<3 g/day	≥3 - <20 g/day	≥20 g/day
Fish (not fried)	<13 g/day	13 g/day	>13 g/day
Whole grains	<30 g/day	≥30 - <90 g/day	≥90 g/day
Poultry	<14 g/day	≥14 - <29 g/day	≥29 g/day
Olive oil	<15 g/day	≥15 - <30 g/day	≥30 g/day
Butter and stick margarine	≥10 g/day	>5 - <10 g/day	≤5 g/day
Full-fat cheese	≥30 g/day	>9 - <30 g/day	≤9 g/day
Red and processed meat	≥100 g/day	>43 - <100 g/day	≤43 g/day
Wine	>100 ml/d	NA	≤100 ml/d
Take out, fried foods and snacks	>3 serving eq/wk	>1 - ≤3 serving eq/wk	≤1 serving eq/wk
Cookies, pastries and sweets	>4 serving eq/wk	>2 - ≤4 serving eq/wk	≤2 serving eq/wk

Supplementary Table 3. Data quality measures and tissue fractions of ^{1}H-MRS data (*n*=88) within the dorsolateral prefrontal cortex.

	Cr SNR	Cr FWHM	Water FWHM	relResA	GM %	WM %	CSF %
Mean ± SD	62.97 ± 11.0	7.87 ± 1.72	8.52 ± 1.85	2.92 ± 1.8	34 ± 6	61 ± 8	4.9 ± 3.7

Cr = Creatin; SNR = signal-to-noise ratio; FWHM = full width at half maximum; GM = grey matter; WM = white matter; CSF = cerebrospinal fluid; SD = standard deviation

Supplementary Table 4. Correlations between lifestyle factors and study variables

Lifestyle factor	Study variable	Rho	P-value	N
**BMI (kg/m²)**	*MIND diet adherence*	-0.14	0.20	88
	Systemic inflammation	0.18	0.10	88
	Neuroinflammation	-0.04	0.72	88
	Cognition	-0.11	0.30	88
**Physical activity (MET-min/week)**	*MIND diet adherence*	0.03	0.82	83
	Systemic inflammation	-0.14	0.20	83
	Neuroinflammation	0.03	0.78	83
	Cognition	-0.11	0.31	83

MET = Metabolic equivalent of task.

Data was corrected for age and sex. Residuals were used for correlation analyses. The correlation between BMI and physical activity was Rho = 0.06, p = 0.61 (n=83).

Supplementary Table 5. Linear model statistics with and without interaction per model

Models with terms	Estimate (b)	SE	CI (lower)	CI (upper)	Std. Estimate (β)	Std. CI (lower)	Std. CI (upper)	t value	Pr(>|t|)
**MODEL 1a: Neuroinflammation ~ age + sex + relResA + MIND adherence + systemic inflammation (PC1)**									
***n*=88**									
R^2 = 0.023; Adj. R^2 = -0.037; Cohens f^2 = 0.023; F-statistic = 0.39; P = 0.86									
(Intercept)	1.30	2.36	-3.40	5.99	0.02	-0.25	0.29	0.55	0.58
Age	-0.02	0.04	-0.09	0.05	-0.07	-0.30	0.16	-0.57	0.57
Sex (male)	-0.09	0.34	-0.77	0.59	-0.06	-0.55	0.42	-0.26	0.80
relResA (spectral quality)	0.06	0.09	-0.12	0.23	0.07	-0.15	0.29	0.65	0.52
MIND adherence	0.00	0.08	-0.16	0.16	0.00	-0.23	0.23	-0.04	0.97
Systemic inflammation (PC1)	0.12	0.12	-0.11	0.36	0.12	-0.10	0.34	1.05	0.30
**MODEL 1b: Neuroinflammation ~ age + sex + relResA + MIND adherence + systemic inflammation (PC1) + MIND adherence:systemic inflammation (PC1)**									
***n*=88**									
R^2 = 0.074; Adj. R^2 = 0.006; Cohens f^2 = 0.080; F-statistic = 1.09; P = 0.38									
(Intercept)	1.39	2.31	-3.21	5.99	-0.01	-0.27	0.26	0.60	0.55
Age	-0.03	0.04	-0.10	0.05	-0.08	-0.31	0.14	-0.73	0.47
Sex (male)	-0.10	0.33	-0.76	0.57	-0.07	-0.54	0.40	-0.29	0.77
relResA (spectral quality)	0.07	0.09	-0.10	0.24	0.09	-0.13	0.31	0.84	0.41
MIND adherence	0.02	0.08	-0.14	0.17	0.03	-0.20	0.25	0.23	0.82
Systemic inflammation (PC1)	1.11	0.48	0.16	2.05	0.13	-0.09	0.35	2.32	0.02
MIND adherence: Systemic inflammation (PC1)	-0.11	0.05	-0.22	-0.01	-0.22	-0.42	-0.01	-2.12	0.04
**MODEL 2a: Cognition ~ age + sex + education + MIND adherence + systemic inflammation (PC1)**									
***n*=88**									
R^2 = 0.21; Adj. R^2 = 0.16; Cohens f^2 = 0.27; F-statistic = 4.36; P = 0.001									
(Intercept)	3.41	0.86	1.70	5.13	0.01	-0.23	0.25	3.95	0.00
Age	-0.05	0.01	-0.08	-0.03	-0.45	-0.66	-0.24	-4.25	0.00
Sex (male)	-0.02	0.11	-0.24	0.21	-0.03	-0.46	0.41	-0.13	0.89
Education level	0.02	0.03	-0.05	0.08	0.05	-0.15	0.25	0.47	0.64
MIND adherence	0.01	0.03	-0.04	0.06	0.03	-0.17	0.24	0.32	0.75
Systemic inflammation (PC1)	-0.02	0.04	-0.10	0.05	-0.06	-0.26	0.14	-0.61	0.55

**MODEL 2b: Cognition ~ age + sex + education + MIND adherence + systemic inflammation (PC1) + MIND adherence:systemic inflammation (PC1)**									
***n*=88**									
R^2 = 0.27; Adj. R^2 = 0.21; Cohens f^2 = 0.36; F-statistic = 4.89; P <0.001									
(Intercept)	3.33	0.84	1.66	4.99	0.04	-0.19	0.28	3.97	0.00
Age	-0.05	0.01	-0.08	-0.03	-0.43	-0.64	-0.23	-4.19	0.00
Sex (male)	-0.02	0.11	-0.24	0.20	-0.03	-0.46	0.39	-0.16	0.87
Education level	0.02	0.03	-0.04	0.08	0.06	-0.13	0.26	0.63	0.53
MIND adherence	0.00	0.03	-0.05	0.05	0.00	-0.20	0.20	0.03	0.98
Systemic inflammation (PC1)	-0.40	0.16	-0.71	-0.09	-0.07	-0.26	0.12	-2.56	0.01
MIND adherence: Systemic inflammation (PC1)	0.04	0.02	0.01	0.08	0.22	0.04	0.40	2.48	0.02
**MODEL 3a: Neuroinflammation ~ age + sex + relResA + MIND adherence + intestinal barrier permeability (PC1)**									
***n*=71**									
R^2 = 0.028; Adj. R^2 = -0.047; Cohens f^2 = 0.028; F-statistic = 0.37; P = 0.87									
(Intercept)	1.04	2.75	-4.45	6.52	0.03	-0.28	0.34	0.38	0.71
Age	-0.02	0.04	-0.10	0.07	-0.05	-0.31	0.20	-0.42	0.68
Sex (male)	-0.13	0.39	-0.91	0.65	-0.09	-0.62	0.45	-0.32	0.75
relResA (spectral quality)	0.08	0.09	-0.10	0.27	0.11	-0.14	0.36	0.89	0.38
MIND adherence	-0.01	0.09	-0.19	0.17	-0.01	-0.26	0.24	-0.11	0.91
Intestinal barrier permeability (PC1)	0.15	0.15	-0.16	0.46	0.12	-0.13	0.37	0.96	0.34
**MODEL 3b: Neuroinflammation ~ age + sex + relResA + MIND adherence + intestinal barrier permeability (PC1) + MIND adherence: intestinal barrier permeability (PC1)**									
***n*=71**									
R^2 = 0.083; Adj. R^2 = -0.003; Cohens f^2 = 0.090; F-statistic = 0.97; P = 0.46									
(Intercept)	1.09	2.69	-4.28	6.46	-0.03	-0.34	0.28	0.41	0.69
Age	-0.02	0.04	-0.11	0.06	-0.07	-0.32	0.18	-0.58	0.56
Sex (male)	0.18	0.41	-0.65	1.00	0.12	-0.44	0.68	0.43	0.67
relResA (spectral quality)	0.09	0.09	-0.09	0.28	0.12	-0.12	0.37	1.00	0.32
MIND adherence	0.02	0.09	-0.16	0.20	0.03	-0.22	0.27	0.21	0.83
Intestinal barrier permeability (PC1)	1.71	0.81	0.09	3.32	0.20	-0.06	0.46	2.11	0.04
MIND adherence: Intestinal barrier permeability (PC1)	-0.17	0.09	-0.34	0.00	-0.28	-0.56	0.00	-1.97	0.05

Supplementary Table 6. Mediation model statistics predicting cognition. SEM-based mediation analysis predicting mediating effect of neuroinflammation within relation between MIND diet adherence:systemic inflammation interaction and cognition (*n*=88).

Mediation model statistics

Path Coefficients

Path	***Coefficient (SE)***	***95% bootstrap CI***	***Standardized coefficient***	***P-value***
**a**: MIND adherence × Systemic inflammation (PC1) → Neuroinflammation (PC1)	-0.115 (0.068)	[-0.269, -0.004]	-0.959	0.09
**b**: Neuroinflammation (PC1) → Cognition composite score (Z)	-0.028 (0.043)	[-0.127, 0.041]	-0.076	0.51
**c'**: MIND adherence × Systemic inflammation (PC1) → Cognition composite score (Z) (direct)	0.040 (0.023)	[-0.011, 0.081]	0.895	0.09

Effect Estimates

Effect type	***Estimate (SE)***	***95% bootstrap CI***	***Standardized estimate***	***P-value***
Direct effect (c’)	0.040 (0.023)	[-0.011, 0.081]	0.895	0.09
Indirect effect (a x b)	0.003 (0.006)	[-0.005, 0.019]	0.073	0.58
Total effect (c)	0.043 (0.022)	[-0.002, 0.084]	0.968	0.05

Supplementary Table 7. Mediation model statistics predicting neuroinflammation. SEM-based mediation analysis predicting mediating effect of systemic inflammation within relation between intestinal barrier permeability and neuroinflammation within the low (*n*=35) and high (*n*=36) MIND diet adherence group based on median split.

**Mediation model statistics**	**Low MIND group (≤8.75, *n*=35)**				**High MIND group (>8.75, *n*=36)**			
**Path Coefficients**								
Path	***Coefficient (SE)***	***95% bootstrap CI***	***Standardized coefficient***	***P-value***	***Coefficient (SE)***	***95% bootstrap CI***	***Standardized coefficient***	***P-value***
**a:** Intestinal permeability (PC1) → Systemic inflammation (PC1)	0.771 (0.253)	[0.264, 1.272]	0.597	0.002	0.498 (0.213)	[0.123, 0.975]	0.452	0.02
**b**: Systemic inflammation (PC1) → Neuroinflammation (PC1)	0.553 (0.230)	[0.155, 1.055]	0.467	0.02	-0.129 (0.174)	[-0.518, 0.176]	-0.140	0.46
**c'**: Intestinal permeability (PC1) → Neuroinflammation (PC1) (direct)	0.329 (0.417)	[-0.391, 1.204]	0.215	0.43	-0.046 (0.142)	[-0.295, 0.277]	-0.045	0.75
**Effect Estimates**								
Effect type	***Estimate (SE)***	***95% bootstrap CI***	***Standardized estimate***	***P-value***	***Estimate (SE)***	***95% bootstrap CI***	***Standardized estimate***	***P-value***
Direct effect (c’)	0.329 (0.417)	[-0.391, 1.204]	0.215	0.43	-0.046 (0.142)	[-0.295, 0.277]	-0.045	0.75
Indirect effect (a x b)	0.427 (0.212)	[0.072, 0.891]	0.279	0.04	-0.064 (0.096)	[-0.298, 0.081]	-0.063	0.51
Total effect (c)	0.756 (0.493)	[-0.125, 1.716]	0.494	0.13	-0.110 (0.134)	[-0.379, 0.160]	-0.109	0.41

Supplementary figures

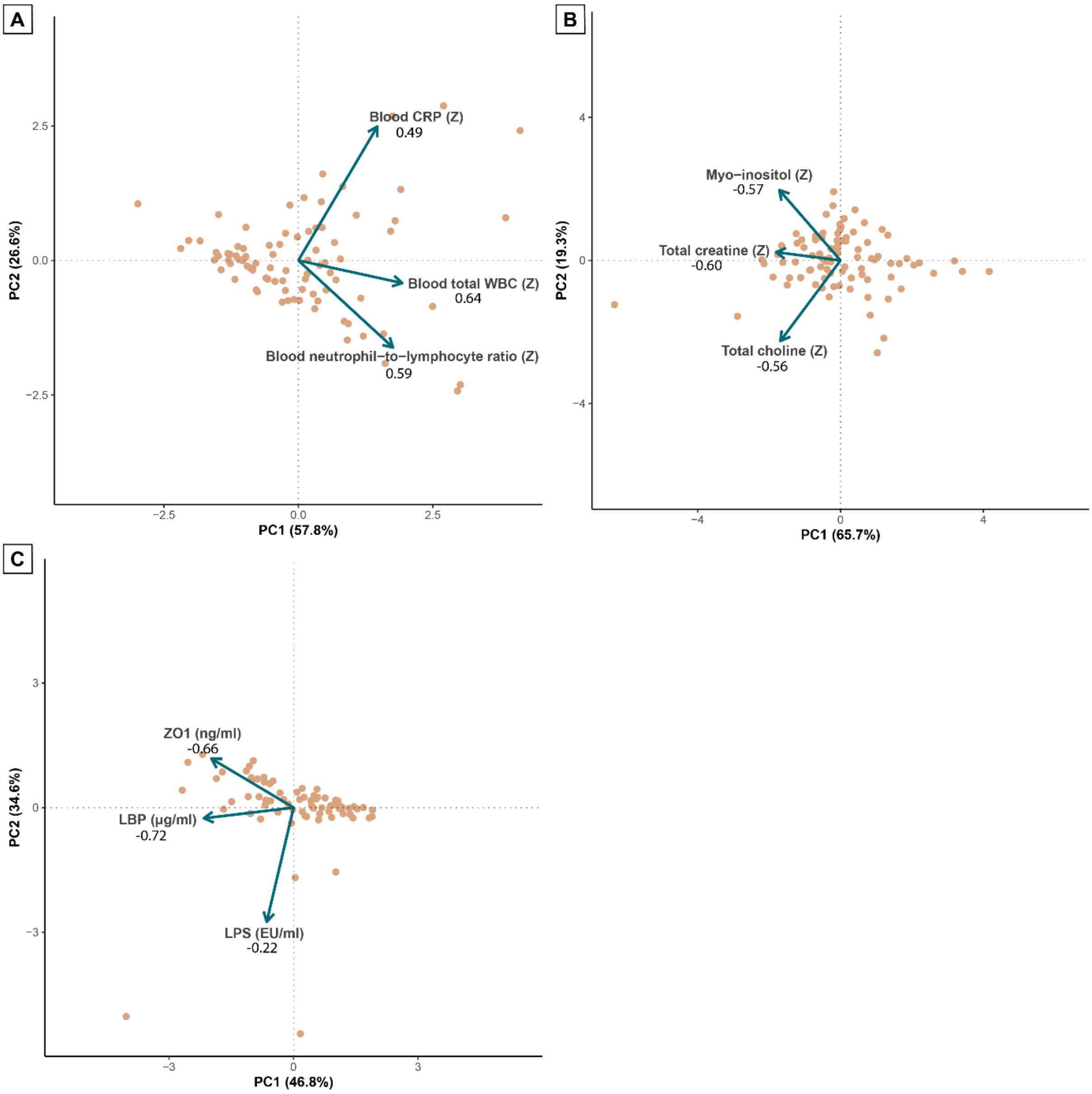

Supplementary Figure 1. PCA plots. *Principal Component Analysis plots of systemic inflammation (n=88) (**A**), neuroinflammation (n=88) (**B**), and intestinal barrier permeability (n=71) (**C**). PC1 loadings are shown below each variable. LPS PC1 loadings were lower on compared to zonulin and LBP for intestinal barrier permeability, probably due to a few extreme outliers.*

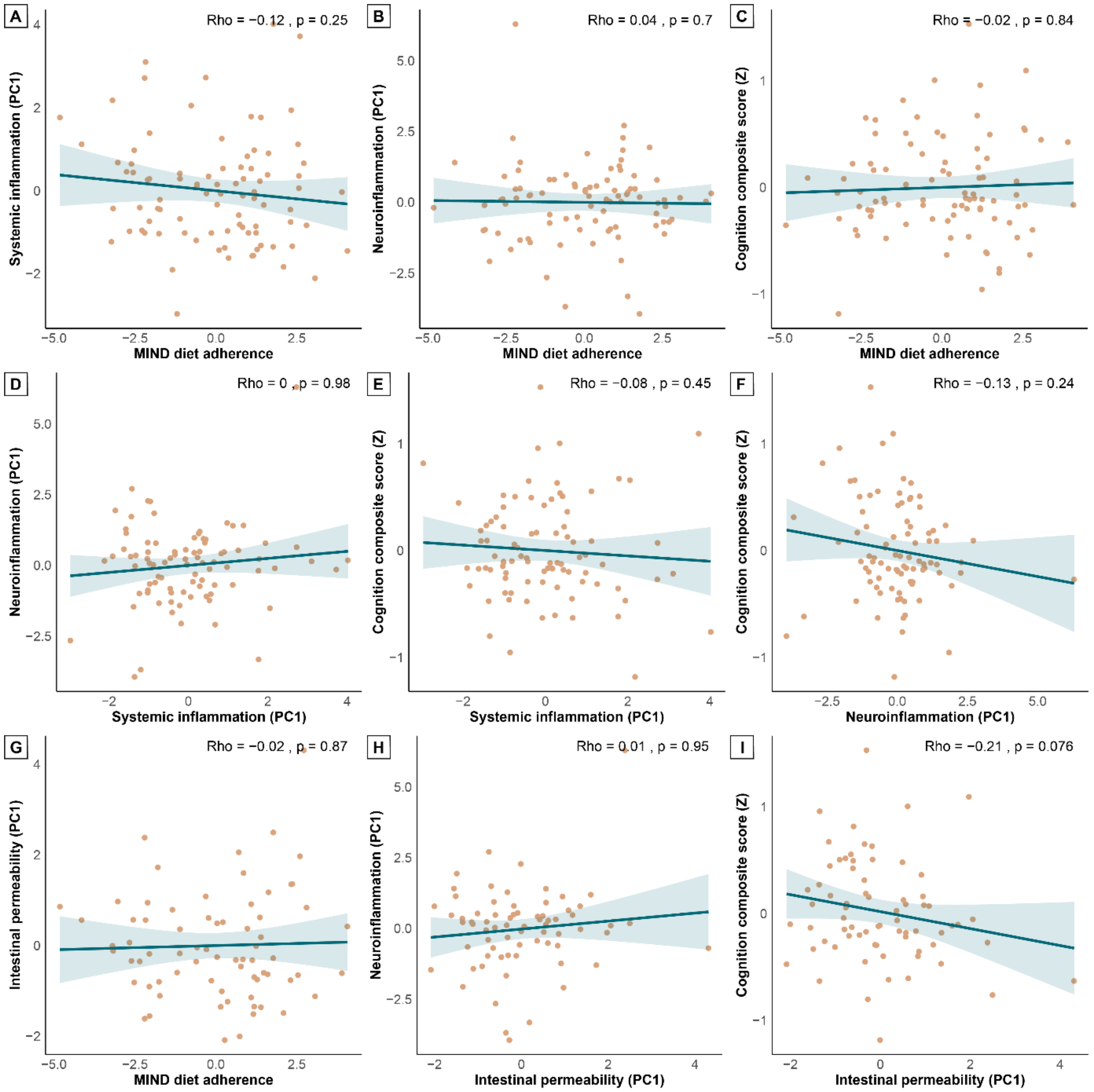

Supplementary Figure 2. Pairwise Spearman correlation scatterplots for MIND diet adherence, systemic inflammation, neuroinflammation, cognition, and intestinal barrier permeability. *Scatterplots showing correlations between MIND diet adherence and systemic inflammation (n=88) (**A**), MIND diet adherence and neuroinflammation (n=88) (**B**), MIND diet adherence and cognition composite score (n=88) (**C**), systemic inflammation and neuroinflammation (n=88) (**D**), systemic inflammation and cognition composite score (n=88) (**E**), neuroinflammation and cognition composite score (n=88) (**F**), MIND diet adherence and intestinal barrier permeability (n=71) (**G**), intestinal barrier permeability and neuroinflammation (n=71) (**H**), and intestinal barrier permeability and cognition composite score (n=71) (**I**).*

Orange dots represent individual subjects. Trendline and confidence interval are displayed in blue. Spearman's rho and p-value are reported top right. Data was corrected for age, sex, relResA/spectral quality (neuroinflammation), and education levels (cognition). Residuals were used for correlation analyses.

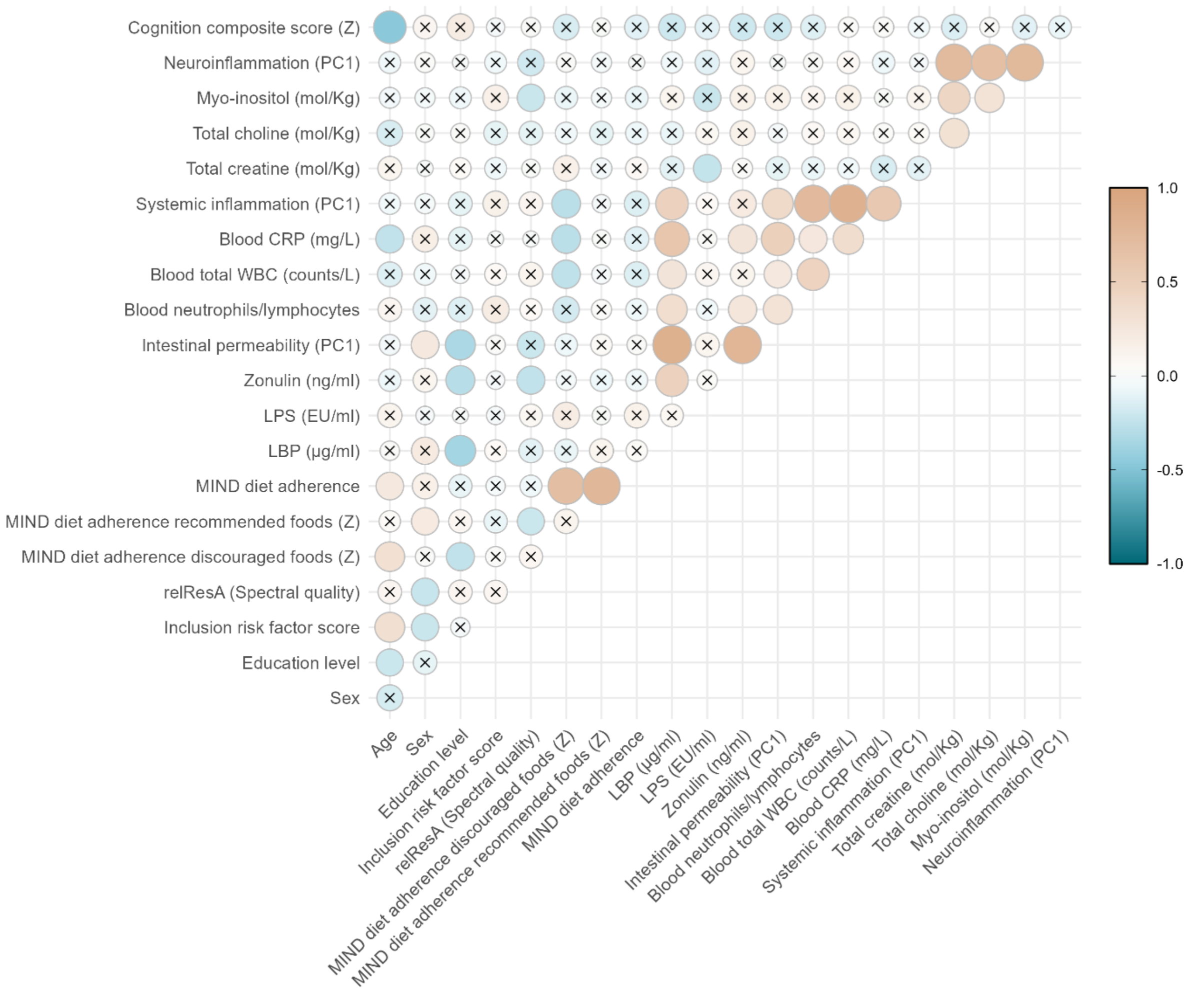

Supplementary Figure 3. Spearman correlation heatmap of all variables, subscores and covariates (n=88). *Heatmap showing correlations between MIND diet adherence, intestinal barrier permeability, systemic inflammation, neuroinflammation, cognition, subscores for each combination score, and other variables of interest/covariates. Orange colours indicate a positive correlation; blue colours indicate a negative correlation. Circle size and color intensity indicate strength of the correlation (Spearman's rho). Absence of a cross indicate significance (p≤0.05) after FDR correction. For intestinal barrier permeability and subscores, correlations include n=71 participants. FDR = false discovery rate.*

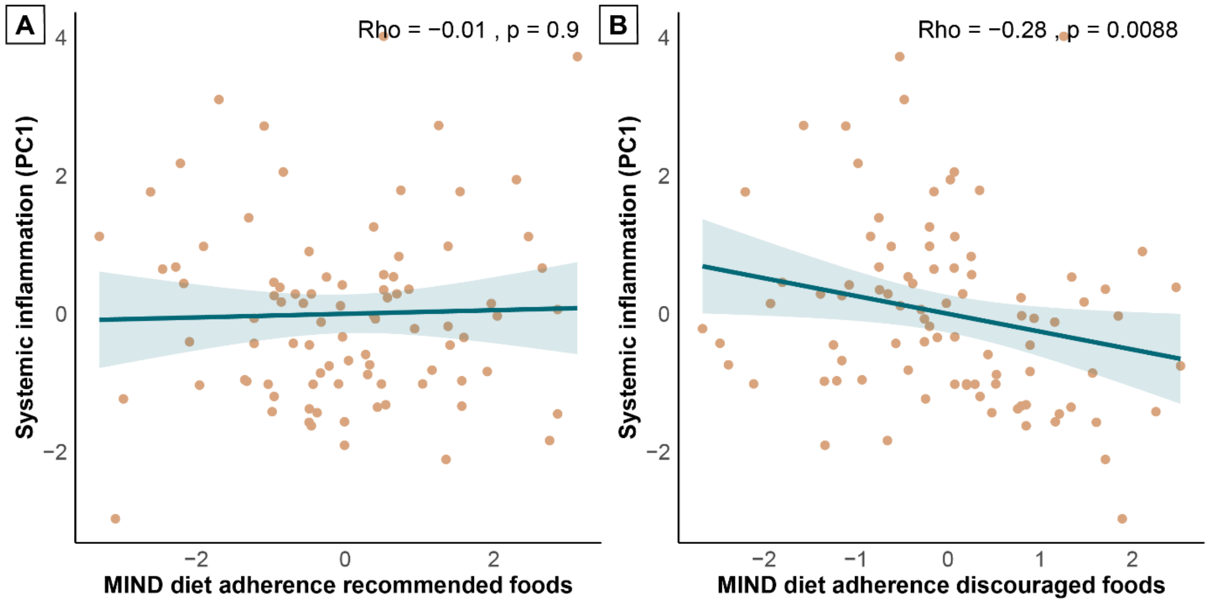

Supplementary Figure 4. Spearman correlation scatterplots for MIND diet adherence to recommended and discouraged foods, and systemic inflammation (n=88). *Scatterplots showing correlations between MIND diet adherence to (consuming) recommended foods and systemic inflammation (**A**), and MIND diet adherence to (limiting) discouraged foods and systemic inflammation (**B**). Orange dots represent individual subjects. Trendline and confidence interval are displayed in blue. Spearman's rho and p-value are reported top right. Data was corrected for age and sex. Residuals were used for correlation analyses.*

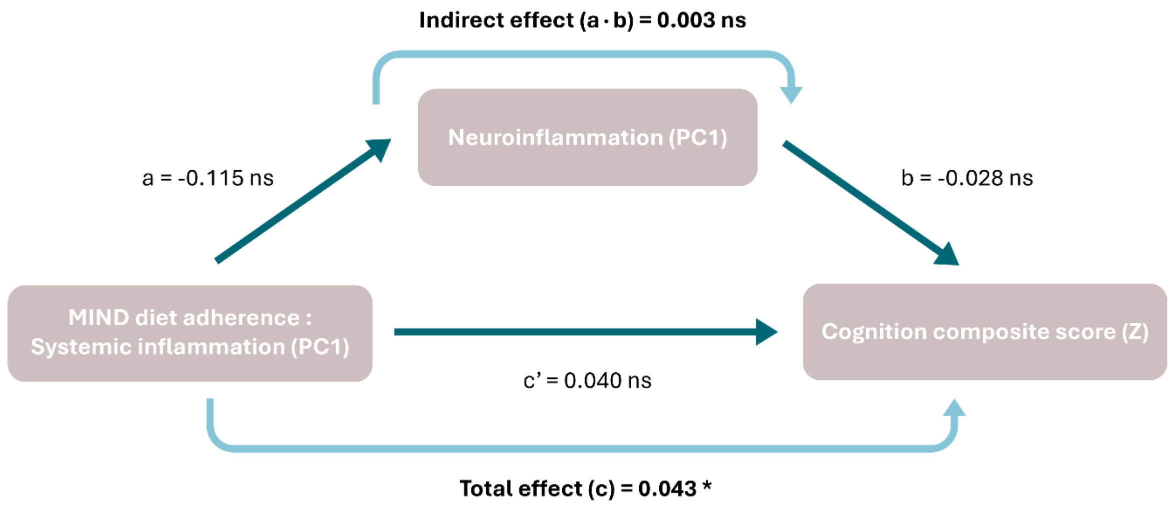

Supplementary Figure 5. Mediation analysis diagram for MIND adherence:systemic inflammation interaction, neuroinflammation, and cognition (n=88). *Diagram showing the mediating role of neuroinflammation in the relation between the interaction term MIND adherence:systemic inflammation and cognition. Path coefficients are reported. Significance is indicated with asterisks (*** = 0.001, ** = 0.01, * = 0.05, ns = non-significant).*
